# Metabolic syndrome and perioperative neurocognitive disorders: epidemiology, mechanisms, and interventions

**DOI:** 10.3389/fnins.2026.1836328

**Published:** 2026-06-05

**Authors:** Jiayi Xie, Bokang Yang, Jinxiang Xie, Chenying Ji, Baoping Zhang, Yuhu Ma, Abdulrahman Khaled Alwesabi, Yatao Liu

**Affiliations:** 1 The First School of Clinical Medicine, Lanzhou University, Lanzhou, Gansu, China; 2 Department of Anesthesiology and Surgery, First Hospital of Lanzhou University, Lanzhou, China

**Keywords:** insulin resistance, metabolic syndrome, neuroinflammation, perioperative neurocognitive disorders, postoperative cognitive dysfunction, postoperative delirium

## Abstract

Perioperative neurocognitive disorders, an umbrella term encompassing preoperative cognitive impairment, acute postoperative delirium, and longer term postoperative neurocognitive disorders, represent significant complications for the growing population of older surgical patients. The rising prevalence of metabolic syndrome, defined by the clustering of abdominal obesity, insulin resistance, hypertension, and dyslipidemia, necessitates a deeper understanding of its impact on the perioperative brain. This comprehensive review elucidates the intricate epidemiological and mechanistic links between metabolic syndrome and the spectrum of cognitive decline. Epidemiologically, we disaggregate the risk profiles of individual components, demonstrating that distinct metabolic phenotypes serve as specific predictors for different phases of impairment. Mechanistically, we propose a sequential pathophysiological cascade where chronic systemic inflammation primes the brain for injury. Surgical stress triggers the failure of a compromised blood brain barrier, leading to the activation of the TLR4/NLRP3 inflammasome and the induction of central insulin resistance. These processes ultimately culminate in mitochondrial energy crises and synaptic degradation. To address these vulnerabilities, we evaluate an integrated perioperative strategy spanning preoperative metabolic optimization, intraoperative management, and emerging pharmacological interventions such as SGLT2 inhibitors and mitochondria targeted antioxidants. Critically, this review identifies a major knowledge gap regarding the absence of dedicated randomized controlled trials targeting the surgical metabolic syndrome population. Ultimately, our findings advocate for a clinical paradigm shift toward phenotype specific metabolic optimization to improve neurocognitive outcomes in these high risk patients.

## 1 Introduction

Commonly affecting memory and attention, perioperative neurocognitive disorders (PND) represent a significant central nervous system (CNS) complication of anesthesia and surgery. With an aging demographic and an increasing number of surgical procedures, PND has become a pressing public health issue. Its impact is extensive: it not only hinders postoperative recovery and extends hospital stays but also drives up healthcare burdens and negatively impacts long-term survival. PND represents a spectrum of cognitive impairments classified by temporal evolution and severity, encompassing acute postoperative delirium (POD), delayed neurocognitive recovery within 30 days after surgery, and mild to major neurocognitive disorders persisting beyond 12 months. Crucially, these distinct entities within the PND spectrum must be clearly differentiated in clinical and diagnostic contexts. POD is an acute, often fluctuating disturbance of consciousness and attention that typically manifests within the first few days after surgery. In contrast, POCD represents a more subtle, subacute-to-chronic decline in specific cognitive domains, such as memory and executive function, emerging weeks to months postoperatively. Because POD and POCD differ fundamentally in their time course, underlying neuropathological mechanisms, and baseline risk associations, merging them obscures specific clinical insights. Therefore, to ensure epidemiological accuracy, this review systematically disaggregates their specific risk profiles when evaluating the impact of metabolic vulnerabilities.

With changes in global population structure, the metabolic profile of surgical populations is undergoing a dramatic transformation. According to the harmonized definition jointly issued by the International Diabetes Federation (IDF), American Heart Association (AHA), and other organizations, the core features of metabolic syndrome (MetS) include central obesity, elevated triglycerides, reduced high-density lipoprotein cholesterol (HDL-C), elevated blood pressure, and elevated fasting glucose (Alberti et al., 2009). Currently, robust epidemiological data estimate that approximately 20–25% of the global adult population suffers from MetS (Saklayen, 2018). Geo-epidemiological data indicate that MetS has reached pandemic proportions globally, with prevalence rates ranging from 20% to over 40% across different regions.

The clinical significance of these epidemiological findings is particularly pronounced in the context of surgical care for aging populations. We are no longer just dealing with isolated cases of high blood sugar or hypertension in the wards; we are looking at a vast demographic entering the operating room with their neurological reserve already heavily depleted by MetS. When the acute stress of surgery and anesthesia is superimposed on a brain subjected to chronic metabolic inflammation, the risk of severe perioperative neurocognitive decline is significantly elevated. To address these clinical challenges, this review aims to systematically evaluate the impact of MetS on perioperative brain health. Our primary exposure of interest is the strictly defined metabolic syndrome as an integrated pathological entity. However, recognizing that high-quality epidemiological evidence treating MetS as a holistic syndrome in the perioperative setting remains relatively limited, we strategically expand our scope to systematically dissect its individual core diagnostic components. By outlining the clinical links of both the overarching syndrome and its specific components to PND, we map the biological damage from insulin resistance to blood-brain barrier collapse and highlight critical interventions to protect the surgical brain.

## 2 Literature search strategy

To ensure a comprehensive and transparent review of the current evidence, a systematic literature search was conducted. We queried major electronic databases, including PubMed/MEDLINE, Embase, Web of Science, and the Cochrane Library, for articles published from January 2000 to January 2026. The search strategy utilized a combination of Medical Subject Headings (MeSH) and free-text keywords, constructed with Boolean operators: (“Metabolic Syndrome” OR “Metabolic Syndrome X” OR “Insulin Resistance” OR “Hyperglycemia” OR “Obesity” OR “Adiposity” OR “Hypertension” OR “Dyslipidemia”) AND (“Perioperative Neurocognitive Disorders” OR “Postoperative Delirium” OR “Postoperative Cognitive Dysfunction” OR “POCD” OR “POD” OR “Cognitive Impairment” OR “Cognitive Decline”) AND (“Perioperative” OR “Postoperative” OR “Surgery” OR “Anesthesia”). The inclusion criteria for the selected literature were: (i) peer-reviewed articles, including prospective and retrospective cohort studies, randomized controlled trials, systematic reviews, and meta-analyses; (ii) studies specifically investigating the association between metabolic syndrome (or its individual components) and neurocognitive outcomes following surgical procedures; and (iii) foundational basic science research utilizing established animal models to explore the underlying molecular mechanisms (e.g., neuroinflammation, blood-brain barrier disruption, and bioenergetic failure) within the perioperative context. The exclusion criteria were: (i) non-peer-reviewed preprints or conference abstracts with insufficient data; (ii) studies focusing solely on neurodegenerative diseases (e.g., Alzheimer’s disease) without a surgical or anesthetic context; and (iii) articles not published in the English language. Two independent authors conducted the initial screening of titles and abstracts, followed by a full-text review to determine the final inclusion of the referenced literature.

## 3 Clinical epidemiological evidence linking MetS and PND

The link between MetS and PND is no longer just a theory; it is backed by massive data. According to a large-scale meta-analysis involving over 5.7 million subjects published newly in 2025, the overall relative risk of cognitive impairment in patients with MetS increased by 34% (95% CI: 1.25–1.43) (Azami et al., 2025). In the perioperative setting, this translates to a significantly elevated clinical risk: the incidence of POD increases by 1.85 times, and their likelihood of facing long-term POCD at 3 months postoperatively is nearly 10% (Feinkohl et al., 2023).

The neurotoxicity of MetS has been validated across different types of surgeries. In non-cardiac surgery, a prospective study confirmed that MetS is an independent predictor of postoperative cognitive dysfunction, indicating that the systemic metabolic disorders it represents significantly weaken the brain’s reserve capacity against surgical stress (Hudetz et al., 2011a). In cardiac surgery, which is more sensitive to hemodynamic fluctuations, patients with MetS who undergo cardiopulmonary bypass have a higher incidence of short-term cognitive dysfunction, and their overall cognitive performance is significantly lower than that of non-MetS patients (Hudetz et al., 2011b). This clinical phenomenon has found biological evidence in basic research: a rat model of metabolic syndrome exhibited more severe acute memory impairment after surgery than the normal control group, and this early damage significantly predicted persistent cognitive decline months after surgery (Feng et al., 2013). When MetS overlaps with other geriatric syndromes, its harm presents a malignant synergistic effect. Research on elderly patients undergoing gastric cancer surgery revealed that when MetS coexists with frailty, the risk of severe postoperative complications increases by more than 10-fold compared to patients with isolated MetS or frailty (Jiang et al., 2024). This adverse synergistic effect indicates that a substantial metabolic burden, combined with depleted physiological reserve, is a major determinant of severe postoperative complications.

Moreover, the cognitive risk associated with MetS is not a static threat; it exhibits a clear dose-response relationship. A landmark 28-year follow-up of the Whitehall II cohort study robustly demonstrated this cumulative neurotoxicity, revealing that the risk of incident cognitive decline escalates progressively with every additional metabolic component present (Machado-Fragua et al., 2022).

## 4 Epidemiological relationship between components of MetS and PND

Such findings naturally prompt a deeper clinical question: if these components synergistically erode brain reserve, what specific pathogenic role does each individual factor—such as obesity or diabetes—play in this cascade? While their individual predictive weights for PND vary across different surgical populations, their collective threat stems from a shared biological progression. The following subsections explore these components in an order that mirrors their mechanistic cascade. We begin with the upstream metabolic triggers, specifically central obesity and insulin resistance. We then transition to the subsequent downstream vascular consequences, including hypertension and dyslipidemia. This structural narrative maps the evolution of the syndrome from initial adipose dysfunction to systemic vascular compromise, highlighting the progressive cascade that ultimately targets the vulnerable brain.

### 4.1 Obesity

Previous studies have not reached a unified conclusion on whether obesity is an independent risk factor for PND. If we look strictly at body mass index (BMI) as a standalone metric, simple obesity often fails to emerge as a statistically significant risk factor for POCD delayed POCD in older adults (Feinkohl et al., 2016). Studies in patients undergoing hip replacement surgery even suggest that a higher BMI might confer a survival or cognitive advantage (Zhu et al., 2014). Whether analyzing BMI as a categorical or continuous variable, simple obesity has not shown a statistically significant correlation with the risk of developing POCD in elderly patients (Feinkohl et al., 2016). Research in other surgical fields has observed a protective effect of being overweight on postoperative survival rates (Mullen et al., 2009). This inconsistency arises because BMI merely reflects total body mass, failing to distinguish between benign lean tissue and metabolically dysfunctional fat depots. With deepening research, it has been confirmed that the key determinant of brain cognitive prognosis is not the quantity of fat, but the metabolic quality of the fat. High-quality evidence confirms a definite link between specific obesity phenotypes and subacute cognitive decline. A direct link between POCD and obesity has been explicitly established in recent systematic evaluations of major surgeries, confirming that obese patients face a significantly higher risk of subsequent neurocognitive deficits driven by systemic inflammation and endothelial disruption (Burns et al., 2023).

In a large multicenter prospective cohort study by the BioCog consortium, researchers included preoperative BMI in their analysis and found that for every 1 kg/m^2^ increase, the risk of POCD at 3 months postoperatively increased by approximately 9% (Feinkohl et al., 2023). A similar, albeit subtler, trend appears in gastrointestinal surgeries, where excess weight continues to act as a significant indicator of cognitive vulnerability, provided the metabolic context is considered (He et al., 2019). The impairment of cognition by obesity is not unidirectional but features a significant bidirectional interaction. A patient’s cognitive baseline shortly after surgery actually predicts how well they will manage their weight up to 3 years later (Spitznagel et al., 2014). This suggests that the neurocognitive deficits widely prevalent in obese populations may significantly reduce the long-term benefits of surgical treatment by weakening the patient’s behavioral compliance or self-management abilities.

Current high quality evidence suggests that metabolically adverse adipose tissue is the phenotype most closely linked to perioperative cognitive vulnerabilities. Rather than mere physical mass, this dysfunctional tissue acts as a potent source of systemic inflammation and adipokine imbalance, which directly compromises the blood-brain barrier and triggers neuroinflammation during the perioperative period. The primary threat to the surgical brain is not the physical weight itself, but the systemic metabolic derangements that accompany it, most notably the concurrent development of insulin resistance and hypertension (Newman et al., 2001). Ultimately, it is this specific metabolically adverse phenotype, rather than BMI or anatomical distribution alone, that serves as the definitive driver of neurocognitive decline.

### 4.2 Hyperglycemia

The brain and neural tissues mainly rely on glucose as an energy substrate; any alteration in carbohydrate metabolism directly affects brain functional outputs, including cognition, executive function, and memory (Sebastian et al., 2023). Within the framework of MetS, hyperglycemia is a core diagnostic component that reflects the state of glucose metabolic dysfunction, encompassing both impaired fasting glucose and overt clinical diabetes. Brain regions, such as the hippocampus, are highly sensitive to the local glucose metabolism abnormalities inherent to diabetes, which can lead to enhanced neuronal synaptic reorganization and astrogliosis (Magariños and McEwen, 2000; Saravia et al., 2002). To address the concerns of clinical heterogeneity, it is essential to evaluate the impact of hyperglycemia on acute POD and delayed POCD independently.

Regarding acute outcomes, perioperative hyperglycemia, particularly when manifested as clinical diabetes, serves as a potent independent driver for POD. Orthopedic cohorts provide some of the most compelling evidence. Large-scale analyses of total joint arthroplasties identify diabetic hyperglycemia as a dominant driver of postoperative delirium, surpassing even hypertension in risk intensity (Zhao et al., 2022). A systematic review from 2025 further confirmed that comorbid diabetes significantly increases the incidence of POD in elderly patients undergoing total joint arthroplasty (Ni et al., 2025). Furthermore, the severity of glycemic dysregulation is closely related to acute cognitive prognosis. Prospective data indicate that patients with insulin-dependent diabetes suffer from significantly higher rates of early POD than those managing their blood glucose without insulin (Ntalouka et al., 2022). Parallel to these acute risks, chronic hyperglycemia significantly elevates the vulnerability to subacute and chronic cognitive deficits (POCD). A recent systematic review confirms that diabetic patients face a 1.44-fold higher overall risk of developing delayed POCD compared to their normoglycemic peers, a threat that escalates sharply in populations over 65 (Liu H. et al., 2025). The neurotoxic impact of diabetes varies significantly depending on the surgical context. In non-cardiac surgery, the risk of POCD in diabetic patients increased by more than twofold—an increase even higher than in cardiac surgery, suggesting that under the high-trauma background of cardiac surgery, the specific effects of metabolic factors may be partially masked, while they are more prominent in non-cardiac surgery (Liu H. et al., 2025). For hip fracture surgery, preoperative hyperglycemia actually surpasses chronological age as a strong predictor of long-term cognitive decline at 1 year (Coviello et al., 2025). Similarly, in vascular surgeries, diabetic patients undergoing carotid endarterectomy show substantially steeper cognitive drops at both 3 and 12 months postoperatively (Sándor et al., 2025).

The pattern of hyperglycemia-induced cognitive impairment may also exhibit time specificity. While elevated preoperative HbA1c, a marker of long-term glycemic control, strongly predicts delayed neurocognitive recovery at 30 days postoperatively, this cognitive gap often closes by 6 months (van Zuylen et al., 2022). This time-specific pattern implies a critical clinical window, suggesting that certain metabolism-induced brain injuries might remain reversible if managed appropriately.

### 4.3 Hypertension

The epidemiological association between hypertension and perioperative cognitive decline initially appears inconsistent, largely because this relationship is confounded by population heterogeneity. Specifically, a systematic review and meta-analysis incorporating 24 studies showed that, upon pooling all populations, there was no statistically significant association between preoperative hypertension and the overall risk of delayed POCD (Feinkohl et al., 2017). Furthermore, a comprehensive umbrella review indicated that while factors like diabetes are supported by suggestive evidence, hypertension does not currently meet the criteria for “convincing” (Class I) or “highly suggestive” (Class II) evidence as an independent risk factor (Travica et al., 2023).

However, this apparent lack of association in broad pooled analyses masks significant vulnerabilities tied to specific surgical contexts and distinct cognitive outcomes. When data are disaggregated, the neurotoxic impact of hypertension depends heavily on the nature of the surgical stressor and the temporal phase of the cognitive impairment. Regarding acute postoperative outcomes, specifically POD, hypertension functions as a robust predictor of risk. Large-scale meta-analyses demonstrate that comorbid hypertension significantly increases the relative risk of developing immediate delirium, particularly in procedures sensitive to hemodynamic shifts such as major orthopedic (Zhao et al., 2022) or cardiac surgeries (Shirvani et al., 2020). Conversely, the influence of hypertension on delayed neurocognitive recovery is more context-dependent. While it may not emerge as a universal risk factor in heterogeneous groups, prospective studies identify a history of hypertension as an independent predictor of POCD in specific high-risk cohorts, including elderly patients undergoing major gastrointestinal tumor resections (Li et al., 2021). Beyond global cognitive decline, hypertension may manifest as deficits in specific cognitive domains. For instance, elderly patients with stage I hypertension undergoing elective joint arthroplasty exhibit significantly lower efficiency in alerting and executive control networks on postoperative day seven compared to normotensive individuals (Zhao D. et al., 2020).

The impact of hypertension also extends to perioperative blood pressure management and long-term neurocognitive prognosis. Evidence suggests that the intraoperative use of vasopressors and an increase in postoperative mean arterial pressure serve as independent predictors of new-onset dementia 6–12 months after hip fracture surgery (Neerland et al., 2017). These clinical observations are supported by biological evidence from established animal models. Spontaneously hypertensive rats exhibit more severe learning and memory impairment after anesthesia and surgery compared to normotensive controls, potentially due to mitochondrial dysfunction driven by the downregulation of mitochondrial uncoupling protein 2 (Liu L. et al., 2025). Ultimately, the role of hypertension is modulated by multiple factors, including the type of surgery, the precision of perioperative blood pressure control, and the baseline physiological reserve of the patient.

### 4.4 Dyslipidemia

For years, the role of circulating lipids in perioperative neurocognitive disorders remained a subject of intense clinical debate. Early umbrella reviews concluded that the overall evidence supporting general dyslipidemia as an independent trigger for delayed POCD was insufficient (Travica et al., 2023). Drilling down into specific lipid markers also failed to establish a statistically significant link between isolated hypercholesterolemia and the risk of POCD (Feinkohl et al., 2018). However, this early consensus missed a critical nuance regarding acute outcomes, as it often overlooked the unique atherogenic lipid profile characteristic of metabolic syndrome. As research shifted focus toward this specific phenotype, defined primarily by elevated triglycerides and depleted high-density lipoprotein (HDL) cholesterol, mounting evidence has firmly repositioned dyslipidemia as a major risk factor for both immediate POD and specific trajectories of POCD.

#### 4.4.1 Hypertriglyceridemia

While general dyslipidemia encompasses various lipid abnormalities, the specific neurotoxic lipid profile of MetS is primarily driven by elevated triglycerides, which impact both acute and delayed cognitive outcomes. Regarding delayed recovery recent prospective cohorts provide compelling proof of this vulnerability. In a 2025 study of patients undergoing laparoscopic gynecological surgeries, preoperative hyperlipidemia, characterized predominantly by hypertriglyceridemia, significantly increased the risk of subacute cognitive impairment, driving the incidence of POCD to 32.7% at 1 month postoperatively compared to just 13.0% in normolipidemic patients (Shen et al., 2025). Conversely, regarding acute outcomes in colorectal cancer surgical populations, a similar trend was observed, and patients with this triglyceride-heavy lipid burden experienced longer durations of postoperative delirium (Zhao et al., 2024). Beyond traditional single metrics, composite indices primarily driven by triglycerides offer superior predictive value for acute disturbances. The triglyceride-glucose index, a surrogate marker for insulin resistance, is significantly positively correlated with the incidence of acute POD following gastric surgery, exhibiting a non-linear “U-shaped” relationship (Yao et al., 2025). This pattern suggests that both extremes of metabolic status are detrimental to cognitive health. While elevated TyG levels reflect the burden of insulin resistance and systemic lipotoxicity, abnormally low TyG levels may serve as a surrogate marker for malnutrition, exhaustion of metabolic reserves, or frailty. In such states, the brain may suffer from a localized deficit in essential lipid substrates and energy supply required for neurorepair and the maintenance of synaptic integrity, thereby lowering the threshold for cognitive impairment following surgical stress (Amirfarzan et al., 2023; Huang et al., 2025).

#### 4.4.2 Low HDL-C

Distinct from the direct metabolic toxicity of elevated triglycerides, the pathological role of low HDL-C in MetS lies in the critical loss of neuroprotection, which predominantly manifests as an acute susceptibility to POD. A newly published 2025 meta-analysis comprising nearly 4,700 patients further cements this association, demonstrating that hyperlipidemia, particularly the low HDL-C phenotype, significantly elevates the risk of acute POD (Qiu et al., 2025). Component analysis reveals a distinct pathogenic pattern in these POD patients: a surge in total cholesterol, triglycerides, and low-density lipoprotein cholesterol, coupled with a severe drop in HDL-C. This pattern perfectly mirrors the classic metabolic syndrome phenotype (Qiu et al., 2025). A study on patients undergoing joint replacement surgery reached a similar conclusion, identifying low levels of HDL-C as an independent risk factor for immediate POD (Lin et al., 2022). This suggests that the anti-inflammatory and amyloid-clearance properties of HDL may play a critical role in this process, and its depletion directly leaves the surgical brain vulnerable to acute perioperative inflammatory storms (Palmer et al., 2019).

In the hemodynamically sensitive realm of cardiac surgery, an elevated LDL/HDL ratio not only anticipates POD but also independently predicts the onset of acute postoperative visual and auditory hallucinations (Abu Khadija et al., 2026). Although early evidence regarding broad dyslipidemia was confounded, recent high-quality studies consistently demonstrate that the specific metabolic syndrome lipid profile, characterized by high triglycerides driving both POD and POCD alongside low HDL-C driving acute POD, is a major and modifiable risk factor for distinct phases of perioperative cognitive decline.

To provide a clear overview of the current literature, a detailed summary of the epidemiological evidence that distinguishes between the holistic syndrome and its individual components is presented in Table 1.

**TABLE 1:** Summary of epidemiological evidence linking metabolic syndrome and its individual components to perioperative neurocognitive disorders.

MetS component	Predictor	Surgery type	Sample size (N)	Cognitive outcome	Follow-up	Effect size (OR/RR, 95% CI)/key findings	Original reference
MetS	MetS	Mixed cohort (Age ≥ 65)	765	POD	7 days	OR = 1.85 (1.26–2.70)	Feinkohl et al., 2023
	MetS	Non-cardiac surgery	90 (60 surgical + 30 controls)	POCD	1 month	Significant↑ (17/30 vs. 8/30, *P* < 0.02)	Hudetz et al., 2011a
Obesity	BMI	Mixed surgical cohorts	1,432	POCD	Variable	RR = 1.27 (0.95–1.70, *P* = 0.10)	Feinkohl et al., 2016
	BMI	Carotid endarterectomy	585	Early cognitive dysfunction (eCD)	24 h	Significant↑ (127/145 eCD cases were obese)	Heyer et al., 2015
Hyperglycemia	Elevated HbA1c	Cardiac surgery	1064	POD	5 days (twice daily)	OR = 1.27 (1.16–1.39)	Kotfis et al., 2019
	Type 2 diabetes	Non-cardiac surgery	228	POD & dNCR	4 days and 9 months	T2DM significantly increased the risk of NCD up to 9 months post-op *P* < 0.05	Ntalouka et al., 2022
	Intraoperative hyperglycemia	Non-cardiac surgery	87	POD	7 days	OR = 3.86 (1.13–39.49)	Windmann et al., 2019
	Elevated first glucose	Cardiac surgery	855	POCD	∼4 years	HR = 1.16 (1.13–1.20)	Zhang et al., 2014
	Diabetes mellitus	Cardiac surgery	44	POCD	12 months	Significant ↑ (*P* = 0.024)	Florido-Santiago et al., 2023
	Diabetes mellitus	Carotid surgery	104	POCD	3 and 12 months	Significant ↓ (*p* = 0.042 at 12 m)	Sándor et al., 2025
	Diabetes mellitus/HbA1c	Non-cardiac	102	DNR & POCD	7 days and 6 months	DM increased DNR risk (*p* = 0.031); HbA1c negatively correlated with Δcognition (*p* = 0.003)	van Zuylen et al., 2022
	Insulin resistance (TyG Index)	Gastric surgery	819	POD	ICU stay	OR = 1.25 (1.01–1.56)	Yao et al., 2025
Hypertension	Intraoperative BPV (CV-MAP > 10%)	Hip fracture	1002	POD	During hospitalization	OR = 1.45 (1.03–2.03)	Yang et al., 2025
	Post-op high MAP and vasopressors	Hip fracture	696	POD	6–12 months	High post-op MAP & peri-op vasopressors associated with dementia	Neerland et al., 2017
	blood pressure ≥ 130 mmHg	Cardiac surgery (CPB)	87	Postoperative neurocognitive decline (NCD)	4 days and 4 weeks	Chronically elevated SBP significantly correlated with RBANS score decline (*P* = 0.03)	Stanley et al., 2025
	Post-op high MAP	Hip fracture	696	New-onset dementia	6–12 months	High post-op MAP was associated with new-onset dementia.	Newman et al., 2001
Dyslipidemia	High TG	Knee/Hip arthroplasty	635	POD	7 days	OR = 2.483 (1.573∼3.918) *P* < 0.001	Lin et al., 2022
	Hyperlipidemia (elevated TG and LDL-C)	Meta-analysis	4,686	POD	Variable	OR = 1.47 (1.13–1.91)	Qiu et al., 2025

## 5 Compounding clinical vulnerabilities: age, frailty, and sex differences

The influence of metabolic syndrome on perioperative neurocognitive disorders is closely tied to the process of accelerated biological aging. Metabolic dysregulation significantly exacerbates inflammaging, which is a state of chronic and sterile inflammation that amplifies natural age-related decline (Franceschi and Campisi, 2014). Specifically, chronic hyperglycemia and lipid toxicity drive the accumulation of senescent cells within the neurovascular unit. These cells develop a senescence-associated secretory phenotype and persistently release pro-inflammatory cytokines. This chronic inflammatory environment primes microglia to mount an exaggerated neurotoxic response following surgical trauma (Palmer et al., 2019).

In addition to inflammatory pathways, metabolic syndrome disrupts the physiological and structural integrity required to maintain baseline cognitive reserve. Chronic metabolic stress leads to endothelial impairment and neurovascular uncoupling, which are functional deficits that mirror the pathology of advanced chronological aging (Toth et al., 2017). These disturbances are often accompanied by microvascular rarefaction and white matter disintegration, effectively depleting the compensatory capacity of the brain. Consequently, when these patients are subjected to standard surgical insults, the lack of vascular resilience makes them more likely to transition from subclinical vulnerability to overt cognitive dysfunction. Consequently, when these patients are subjected to standard surgical insults, the lack of vascular resilience makes them more likely to transition from subclinical vulnerability to overt cognitive dysfunction, leaving the geriatric brain biologically older and less resilient than chronological age alone would indicate.

This process of biological aging also intertwines with the physical aspects of frailty. While frailty and metabolic syndrome independently increase surgical risk, their concurrent presence exerts a cumulative burden on physiological resilience (Jiang et al., 2024). Chronic inflammation induced by metabolic syndrome accelerates the functional decline, mitochondrial dysfunction, and disrupted proteostasis associated with frailty. Conversely, the reduced homeostatic reserve characteristic of frailty diminishes the brain’s capacity to buffer metabolic insults, such as oxidative stress and glycemic dysregulation. This synergistic interaction (Jiang et al., 2024) further compromises cognitive reserve, rendering frail patients with metabolic dysregulation highly susceptible to adverse postoperative neurocognitive outcomes.

Biological sex also acts as a critical modulator of this vulnerability. The pathophysiological trajectory of cognitive impairment associated with metabolic syndrome diverges distinctly between older males and females. Males generally exhibit a higher baseline burden of visceral adiposity and cardiovascular comorbidities, which predominantly drive cerebrovascular risk. In contrast, aging females undergo a major physiological transition post-menopause. The age-related decline in estrogen, a hormone that typically provides anti-inflammatory and neuroprotective effects, interacts negatively with metabolic dysfunction. This loss of estrogenic modulation amplifies systemic inflammaging and increases blood-brain barrier vulnerability (Auvais-Jarvis et al., 2017). Consequently, frail and elderly females with metabolic syndrome represent a distinct high-risk phenotype, highlighting the need for sex-specific and frailty-informed perioperative optimization strategies.

## 6 Mechanisms

### 6.1 Neuroinflammation

Visceral fat is an active endocrine organ, not just a storage site. In MetS, this tissue becomes highly dysfunctional, directly driving systemic inflammation. These peripheral inflammatory signals cross into the central nervous system, keeping the brain in a chronic inflammatory state. This inflammatory shift serves as the primary trigger for PND. At the center of this process are adipocytes, which drive the pathology through three key mediators: adiponectin, leptin, and fatty acids (Stern et al., 2016). Adipokine imbalance mainly manifests as abnormally elevated levels of the pro-inflammatory factor leptin and the relative depletion of the anti-inflammatory factor adiponectin. Leptin typically rises with increasing fat mass and plays a role in promoting inflammation, atherosclerosis, and endothelial dysfunction (Stern et al., 2016; Feinkohl et al., 2024). Research pointed out that the key pathological change in PND patients is not merely elevated leptin levels, but central leptin resistance caused by abnormalities in the soluble leptin receptor (Feinkohl et al., 2024). This resistance strips leptin of its neuroprotective properties, destroying the neurovascular unit (NVU) (Stern et al., 2016; Feinkohl et al., 2024). Conversely, adiponectin could have exerted powerful anti-inflammatory and BBB protection by inhibiting the NF-κB pathway (Stern et al., 2016; Feinkohl et al., 2024). Obesity elevates circulating free fatty acids. Acting as endogenous damage-associated molecular patterns, these lipids infiltrate the blood-brain barrier to trigger central neurotoxicity (Feinkohl et al., 2024). This molecular infiltration is not a random leak, but a result of the systematic collapse of the NVU. For these circulating factors to cause sustained damage, the structural and functional integrity of the NVU must first be compromised.

### 6.2 Blood-brain barrier damage

The structural integrity and functional coupling of the NVU are the physical foundations for maintaining cognitive function. At the anatomical level, MetS induces pathological vascular remodeling and fundamentally weakens endothelial repair capabilities. Studies show that hypertension causes hyaline degeneration, fibrinoid necrosis, and wall thickening in cerebral arterioles, leading to luminal narrowing and vascular stiffening (Ungvari et al., 2021; Santisteban et al., 2023). Insulin-like growth factor 1 receptor (IGF-1R) functional defects in cerebrovascular smooth muscle cells under hypertensive conditions directly impair vascular wall repair capability, making them highly prone to microhemorrhages and barrier leakage (Miller et al., 2024). The obesity component of MetS has been proven to exacerbate cerebral microvascular rarefaction in elderly mice, and this reduction in microcirculatory density directly limits compensatory reserves for cerebral blood flow, forming the anatomical basis for postoperative cognitive decline (Tucsek et al., 2014). Building upon structural damage, the biochemical toxicity induced by MetS further leads to BBB breakdown. Hyperlipidemia can specifically downregulate the expression of Mfsd2a, a key transporter in the BBB, leading to barrier leakage (Zhang et al., 2019). The low HDL-C levels prevalent in MetS disrupt the anti-inflammatory pathway mediated by the SR-BI receptor on the surface of endothelial cells, rendering them unable to inhibit the expression of adhesion molecules and further exacerbating endothelial damage (Huang et al., 2025; Yao et al., 2025). A high glucose environment induces the over-activation of matrix metalloproteinases, degrading the vascular basement membrane matrix and driving a phenotypic shift in blood vessels toward a pro-contractile, pro-inflammatory, and pro-thrombotic state (Prasad et al., 2014; Rempe et al., 2016). Such hyperpermeability facilitates the translocation of peripheral inflammatory mediators and neurotoxins across the blood-brain barrier, directly injuring hippocampal neurons.

MetS not only destroys structures but also causes the deregulation of NVU functions. Structural stiffness shifts the cerebral blood flow autoregulation curve to the right in hypertensive patients, meaning that the patient’s tolerance to hypotension is reduced. During the perioperative period, a blood pressure range that is safe for a normal person may already be dangerously low for a hypertensive patient, causing the cerebral perfusion pressure to fall below the autoregulatory threshold and triggering silent cerebral ischemia (Santisteban et al., 2023; Bright et al., 2026). As a surrogate marker for severe insulin resistance, a high triglyceride-glucose index induces oxidative stress in cerebral microvascular endothelial cells, depleting nitric oxide (NO) bioavailability (Zhang et al., 2022; Zeng et al., 2025). This leads to endothelial dysfunction and decreased smooth muscle reactivity, causing neuronal activity to be unable to trigger a local increase in blood flow. This supply-demand mismatch places synaptic function in a continuous state of energy starvation, ultimately inducing an acute decline in cognitive function (Zeng et al., 2025). Beyond acting as a physical shield, the NVU serves as the brain’s primary clearance route. In healthy states, HDL particles facilitate the trans-BBB efflux of beta-amyloid through the endothelial ABCA1 transporter. The characteristic low HDL levels in MetS cripple this efflux mechanism, driving pathological beta-amyloid deposition within the hippocampus. The influx of systemic toxins and the accumulation of metabolic waste drive the acceleration of perioperative neurocognitive impairment.

### 6.3 Central insulin resistance

As the body’s most metabolically demanding organ, the brain consumes approximately 20–25% of systemic glucose to sustain neuronal signaling (Arnold et al., 2018). In MetS, neuropathology is driven less by systemic glycemic fluctuations and more by the onset of central insulin resistance. This resistance impairs neuronal glucose uptake, leading to a severe bioenergetic deficit that renders it vulnerable to perioperative stress and subsequent PND (Mergenthaler et al., 2013).

Chronic low-grade inflammation in a MetS environment activates stress kinases such as JNK, triggering the inhibitory serine phosphorylation of insulin receptor substrate-1 and culminating in central insulin resistance (De Felice and Ferreira, 2014). This molecular modification severs the downstream conduction of the PI3K/Akt signaling pathway, preventing the glucose transporter GLUT4 from translocating to the cell membrane (De Felice and Ferreira, 2014). This blockade leaves neurons starved of energy despite systemic hyperglycemia, failing to meet the bioenergetic demands of synaptic transmission. Beyond energetic failure, the inhibition of PI3K/Akt disinhibits downstream glycogen synthase kinase-3β (GSK-3β), promoting Tau hyperphosphorylation and neurofibrillary tangle formation—a critical molecular bridge linking metabolic derangement to Alzheimer’s-like neuropathology (De Felice and Ferreira, 2014). Deprived of metabolic substrates, mitochondrial dynamics collapse, resulting in ATP exhaustion and severe oxidative stress. While physiological stress typically recruits the AMPK pathway to initiate mitophagy, the MetS environment uncouples this adaptive mechanism by overactivating the mTOR pathway, thereby arresting autophagic flux (Chen et al., 2023). The resulting accumulation of dysfunctional mitochondria serves as a relentless source of reactive oxygen species, which directly activate the NLRP3 inflammasome to exacerbate synaptic damage (Ward et al., 2019; Wang et al., 2024).

The metabolic collapse in MetS extends beyond neurons to disrupt coupling within the entire NVU. Energy metabolism impairment in hippocampal astrocytes induced by elevated blood glucose directly leads to a significant downregulation of the expression of glutamate transporter-1 on their surface. It is responsible for recycling over 90% of the glutamate in the synaptic cleft. The loss of glutamate transporter-1 leads to abnormal accumulation of glutamate in the synaptic cleft, overactivating NMDA receptors on the postsynaptic membrane, and inducing severe excitotoxicity, which leads to neuronal calcium overload and cell death; this is considered the direct cause of the sharp decline in postoperative cognitive function in MetS patients (Jiao et al., 2024).

Therefore, the perioperative period is not only a phase of inflammatory storm but also a period of energy shortage in the brain, ultimately leading to a sharp decline in postoperative cognitive function.

### 6.4 Synaptic dysfunction

Consequently, the aforementioned cascade of neuroinflammation and central insulin resistance ultimately culminates in severe synaptic structural damage. The convergence of systemic toxic influx and metabolic waste retention exacerbates perioperative neurocognitive decline by triggering a profound immune crisis within the brain parenchyma. Once across the breached barrier, circulating Free fatty acids and inflammatory cytokines act as potent triggers, directly activating Toll-like receptor 4 on hippocampal microglia (Muscat et al., 2024). This activation shifts microglia from a homeostatic state to a pro-inflammatory M1 phenotype, initiating a self-perpetuating cycle of neuroinflammation. At the molecular level, this microglial priming is amplified by the NLRP3 inflammasome. In the context of MetS, reactive oxygen species and mitochondrial dysfunction synergize with Toll-like receptor 4 signaling. This interaction promotes the assembly and activation of the NLRP3 inflammasome. Consequently, this drives microglia into a hyperactive state, exacerbating the pathological engulfment of synaptic structures and resulting in severe neuroinflammation and synaptic loss. This aberrant synaptic pruning, a core driver of metabolic cognitive impairment, can be significantly reversed by NLRP3 inhibition (Ward et al., 2019). Furthermore, recent preclinical models demonstrate that metabolic dysregulation, such as that induced by a high-fat diet, directly exacerbates this neuroimmune response and evokes severe synaptic degradation, further compromising memory consolidation (Mackey-Alfonso et al., 2024).

The peripherally imported inflammatory signals ultimately lead to the disruption of internal signaling pathways within hippocampal neurons. The MetS-driven inflammatory milieu suppresses the deacetylase Sirt1, thereby crippling the synthesis of brain-derived neurotrophic factor (BDNF) through a dual molecular blockade. BDNF is a key molecule for maintaining long-term potentiation; its deficiency directly leads to the collapse of hippocampal synaptic plasticity, rendering the brain unable to cope with the memory stress brought by anesthesia and surgery (Zhao Z. et al., 2020; Ma et al., 2022). On one hand, the downregulation of Sirt1 blocks the phosphorylation of the transcription factor CREB, thereby severing the synthesis pathway of BDNF (Ma et al., 2022). On the other hand, it inhibits the Sirt1/PGC-1α/FNDC5 axis, leading to a decrease in the expression of the key irisin precursor FNDC5, thereby weakening its ability to induce BDNF expression (Zhao Z. et al., 2020).

**FIGURE 1:**
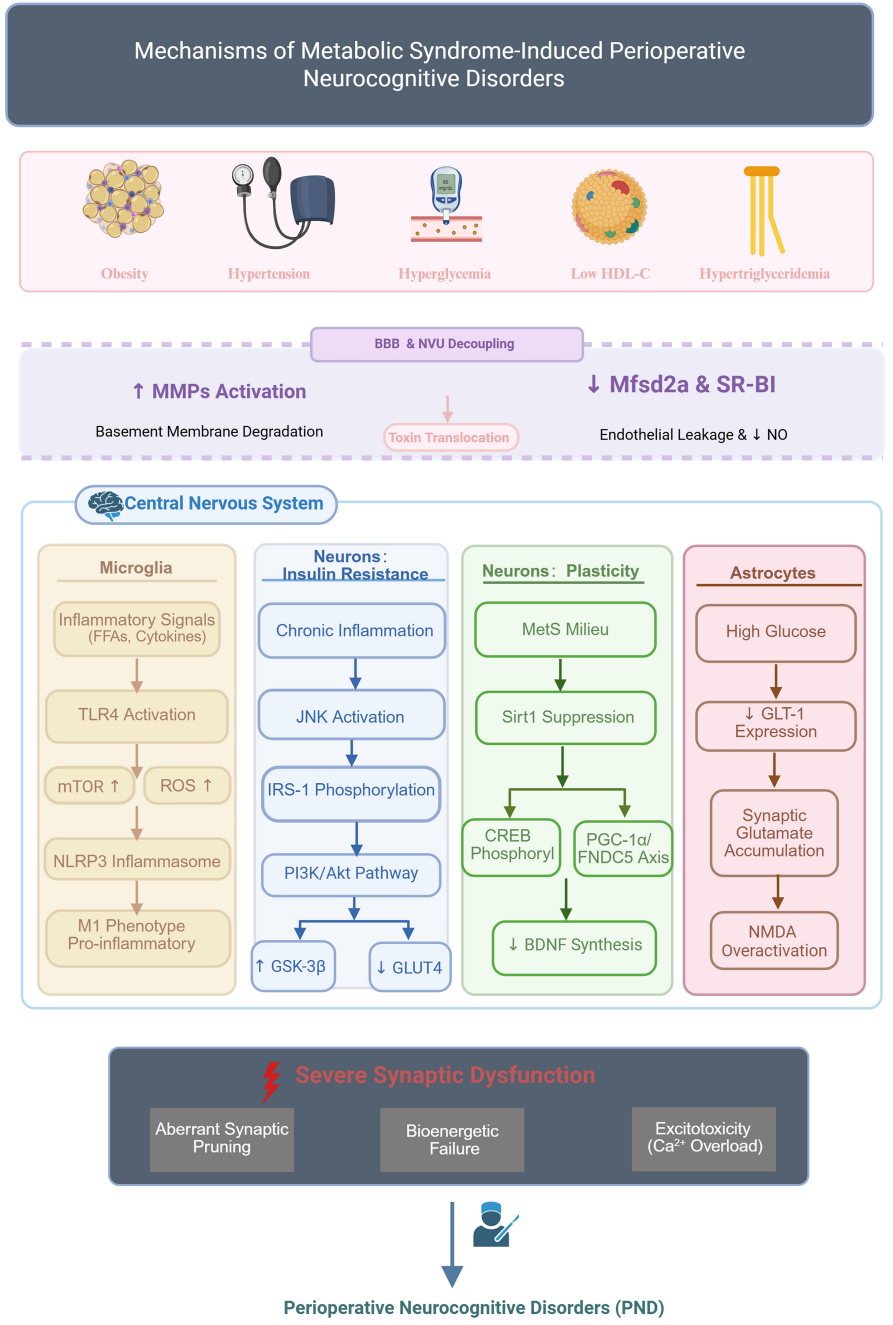
Mechanisms of metabolic syndrome (MetS)-induced perioperative neurocognitive disorders (PND). Figures were created using BioRender.

In summary, the occurrence of PND in patients with MetS is not accidental, but rather a vicious cycle caused by the TLR4/NLRP3 activation—Sirt1/BDNF axis inhibition pathway. Once this vulnerable brain, which is in a subclinical inflammatory state, encounters surgical stress, it easily crosses the compensatory threshold and falls into comprehensive cognitive decline (Figure 1).

## 7 Interventions and prevention

### 7.1 Preoperative metabolic optimization

Rather than a passive waiting period, the preoperative phase presents a critical window for metabolic reprograming and cognitive prehabilitation in the MetS population. Since isolated physical exertion fails to fully reverse cognitive vulnerability, emerging evidence champions cognitive-motor dual-task training—the concurrent execution of aerobic and cognitive challenges (Gheysen et al., 2018). This synergistic modality outperforms singular interventions by robustly stimulating hippocampal BDNF release and restoring synaptic plasticity, directly counteracting the Sirt1/BDNF axis collapse induced by metabolic stress. On the nutritional front, targeted dietary interventions serve as potent systemic anti-inflammatories. Supplementation with specific polyphenols, such as olive leaf extract and punicalagin, directly attenuates metabolic inflammation by inhibiting the NLRP3 inflammasome pathway, thereby halting the immune crisis before surgical trauma occurs (Mikami et al., 2021; Chen et al., 2025). Concurrently, modulating the gut-brain axis via probiotic or postbiotic supplementation fortifies the intestinal barrier; this curtails the systemic translocation of lipopolysaccharide (LPS) and subsequently blunts microglial hyperactivation within the hippocampus (Chunchai et al., 2018). Adopting a Mediterranean diet pattern provides a cost-effective, foundational strategy to sustain these neuroprotective effects through its dense profile of Omega-3 fatty acids and polyphenols (Muscat et al., 2024).

### 7.2 Perioperative anesthetic management

Intraoperatively, anesthetic selection must prioritize neurovascular stability and redox balance. Volatile anesthetics like isoflurane have been shown to exacerbate postoperative cognitive impairment (Yang et al., 2014). Intravenous agents such as propofol or dexmedetomidine offer distinct neuroprotective advantages due to their inherent antioxidant profiles. Given the baseline sympathetic hyperactivity inherent to the MetS phenotype, the administration of dexmedetomidine proves particularly beneficial. Beyond its direct anti-inflammatory properties, it effectively blunts the perioperative sympathetic surge, thereby stabilizing microcirculatory perfusion and shielding the brain from catecholamine-induced neurotoxicity (Xiong et al., 2016). Furthermore, as established previously, the compromised NVU and rightward-shifted cerebral autoregulation curve render MetS patients exceptionally vulnerable to hemodynamic fluctuations. Consequently, relying solely on standard, absolute blood pressure targets poses significant clinical risks. Maintaining intraoperative mean arterial pressure within 80%–100% of the patient’s individual baseline is imperative to sustain cerebral perfusion pressure above this elevated autoregulatory threshold. To further avert the severe bioenergetic deficits characteristic of MetS, multimodal neuromonitoring is essential. Integrating near-infrared spectroscopy for regional cerebral oxygenation with the bispectral index enables the precise, closed-loop titration of anesthetic depth, effectively precluding silent cerebral ischemia and severe supply-demand mismatches during surgery.

### 7.3 Postoperative pharmacological interventions

Postoperative pharmacological management focuses on active neuro-restoration. Repurposing metabolic regulators helps reverse central insulin resistance. In preclinical models, GLP-1 receptor agonists directly suppress hippocampal neuroinflammation and enhance synaptic plasticity (Zhu et al., 2025). Similarly, animal studies suggest that SGLT2 inhibitors like dapagliflozin transcend their systemic glucosuric effects; postoperatively, they actively restore brain mitochondrial function, neutralize oxidative stress, and reinforce the compromised NVU (Kostrzewska et al., 2025). Regarding postoperative statin therapy, early systematic reviews concluded that the clinical evidence for its direct neuroprotective effect against delayed POCD remains insufficient (Feinkohl et al., 2018). Nevertheless, maintaining established statin treatment in the perioperative window is generally advocated to preserve endothelial function and resist ischemic-hypoxic injury, although more robust trials are required to confirm cognitive benefits. Mitochondria-targeted antioxidants (such as SS-31) represent a highly promising translational frontier. In murine models of metabolic stress, by specifically accumulating in the inner mitochondrial membrane, SS-31 clears the postoperative ROS surge and rehabilitates mitochondrial dynamics, effectively halting the progression to cellular apoptosis (Tarantini et al., 2018). Postoperative circadian disruption exacerbates neuroinflammation. Exogenous melatonin supplementation regulates this disturbed biorhythm and acts as an antioxidant. It inhibits neuronal apoptosis via the SIRT1 pathway, improving postoperative sleep quality and cognitive function (Jan et al., 2024).

However, a critical knowledge gap remains. The current enthusiasm for these metabolic reprogrammers and targeted antioxidants in the perioperative setting is predominantly based on preclinical evidence and extrapolation from broad neurodegenerative or cardiovascular studies (Szeto, 2014; Erbil et al., 2022). To date, there is a distinct absence of published randomized controlled trials evaluating the specific cognitive efficacy of GLP-1 agonists, SGLT2 inhibitors, or SS-31 for PND prevention within the surgical MetS cohort. Future clinical investigations must bridge this translational divide to confirm whether these robust preclinical mechanisms translate into tangible neuroprotection for aging surgical patients.

## 8 Conclusion and perspectives

**FIGURE 2:**
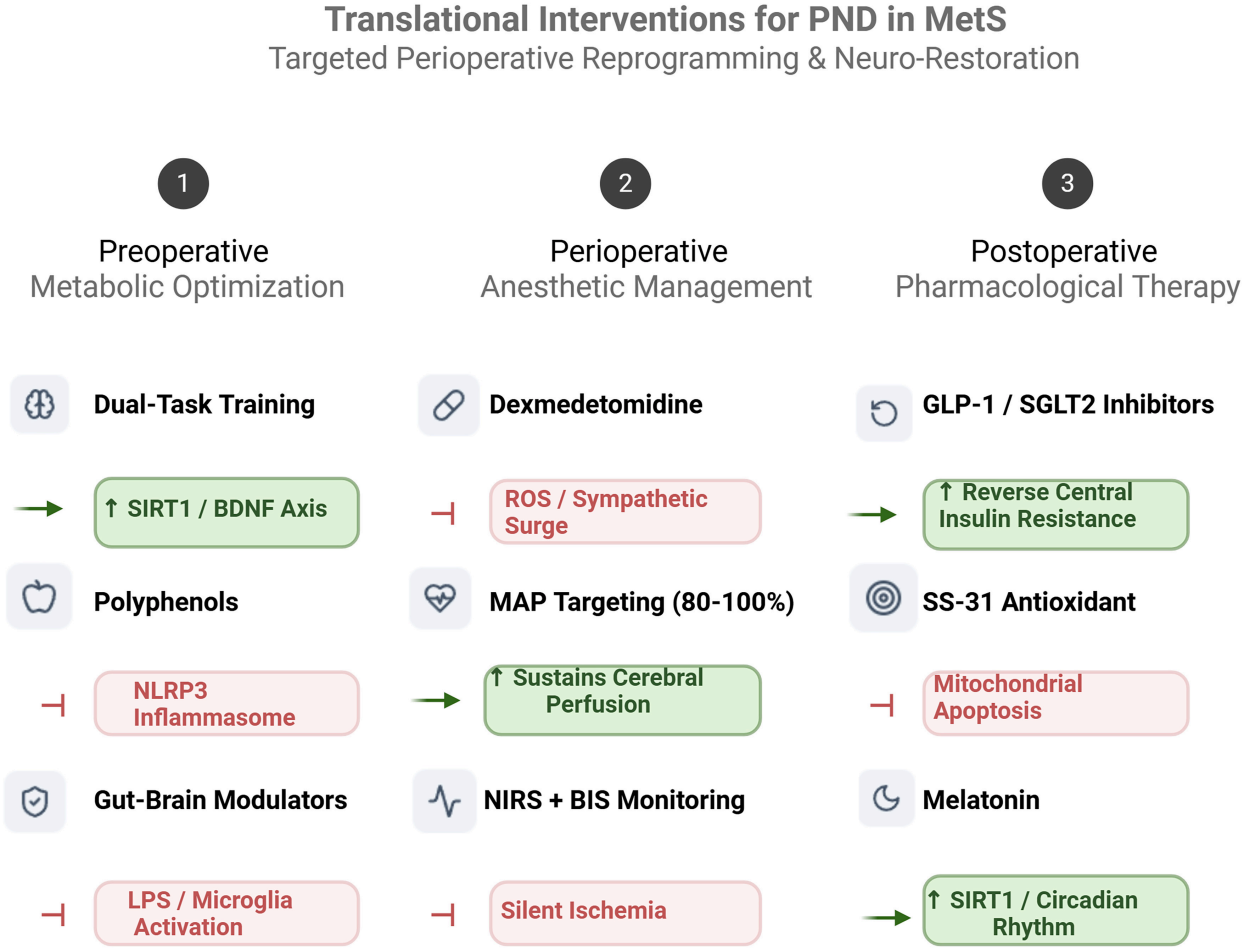
Interventions for PND in MetS. Figures were created using BioRender.

MetS represents a state of systemic metabolic dysfunction that fundamentally amplifies the risk of PND. Rather than a mere summation of its individual components, MetS drives cognitive decline through a synergistic convergence of adipokine imbalance, central insulin resistance, chronic neuroinflammation, and BBB breakdown. Together, these mechanisms establish a profound baseline vulnerability in the surgical brain. Current preoperative evaluations rely heavily on static metabolic indices, frequently failing to capture underlying micro-pathological brain shifts. Future research must prioritize MetS-specific cognitive risk prediction models that incorporate dynamic biomarkers of vascular endothelial damage, axonal injury, and gut microbiota signatures. Such multidimensional models are essential to identify covert high-risk patients whose brain function is already compromised despite seemingly stable systemic metabolism. Standard perioperative management, which primarily targets intraoperative hemodynamic stability, remains insufficient for this population. Interventions must shift toward proactive preoperative metabolic prehabilitation. Given that bioenergetic failure fundamentally drives neurocognitive decline, mitochondria-targeted interventions represent a critical therapeutic frontier (Tarantini et al., 2018). Ultimately, safeguarding the neurocognitive health of surgical MetS patients necessitates a multidisciplinary paradigm led by anesthesiology and integrating endocrinology, nutrition, and rehabilitation (Puri et al., 2023; Figure 2). By optimizing the perioperative window, this comprehensive approach extends beyond immediate surgical safety; it offers a critical opportunity to disrupt the pathological trajectory from metabolic derangement to irreversible cognitive decline, thereby preserving long-term quality of life.

## References

[B1] Abu Khadija H. Abu Hamdeh N. Najajra D. Masu’d M. Dabash A. Zahran A. et al. (2026). Hyperlipidemia and postoperative hallucinations after cardiac surgery: insights from the VAACS Cohort Study. *J. Cardiothorac. Vasc. Anesth*. 40 1155–1170. 10.1053/j.jvca.2025.11.036 41507022

[B2] Alberti K. G. Eckel R. H. Grundy S. M. Zimmet P. Z. Cleeman J. I. Donato K. A. et al. (2009). Harmonizing the metabolic syndrome: a joint interim statement of the International Diabetes Federation Task Force on Epidemiology and Prevention; National Heart, Lung, and Blood Institute; American Heart Association; World Heart Federation; International Atherosclerosis Society; and International Association for the Study of Obesity. *Circulation* 120 1640–1645. 10.1161/CIRCULATIONAHA.109.192644 19805654

[B3] Amirfarzan H. Azocar R. J. Shapeton A. D. (2023). “The Big Three” of geriatrics: a review of perioperative cognitive impairment, frailty and malnutrition. *Saudi J. Anaesth*. 17 509–516. 10.4103/sja.sja_532_23 37779565 PMC10540988

[B4] Arnold S. E. Arvanitakis Z. Macauley-Rambach S. L. Koenig A. M. Wang H. Y. Ahima R. S. et al. (2018). Brain insulin resistance in type 2 diabetes and Alzheimer disease: concepts and conundrums. *Nat. Rev. Neurol*. 14 168–181. 10.1038/nrneurol.2017.185 29377010 PMC6098968

[B5] Auvais-Jarvis F. Bairey Merz C. N. Barnes P. J. (2017). Sex differences in metabolic homeostasis, diabetes, and obesity. *Biol. Sex Differ.* 8:22. 10.1186/s13293-015-0033-y 26339468 PMC4559072

[B6] Azami M. Afraie M. Mohammadzadeh P. Moradkhani A. Shanazari M. Soltanian D. et al. (2025). Association between metabolic syndrome and cognitive impairment: a meta-analysis of analytical observational studies. *Cogn. Neuropsychiatry* 30 127–147. 10.1080/13546805.2025.2503445 40392146

[B7] Bright M. Fanning J. Highton D. (2026). Perioperative blood pressure and neurocognitive disorders after noncardiac surgery: a focused review. *J. Neurosurg. Anesthesiol*. 38 3–9. 10.1097/ANA.0000000000001073 41342779

[B8] Burns C. I. Boghokian A. Soti V. (2023). Obesity and postoperative cognitive dysfunction: a curious association. *Cureus* 15:e42436. 10.7759/cureus.42436 37497308 PMC10368079

[B9] Chen P. Chen X. Zhang H. Chen J. Lin M. Qian H. et al. (2023). Dexmedetomidine regulates autophagy via the AMPK/mTOR pathway to improve SH-SY5Y-APP cell damage induced by high glucose. *Neuromol. Med*. 25 415–425. 10.1007/s12017-023-08745-2 37017880

[B10] Chen P. Lei J. Wang R. Li C. Zhou B. Zhang R. (2025). Ellagitannin component punicalin prevents cognitive impairment by inhibiting metabolic disorders, TLR4/NF-kB/NLRP3 inflammasome signaling, and mitochondrial dysfunction in high-fat diet-fed mice. *Exp. Neurol*. 395:115479. 10.1016/j.expneurol.2025.115479 40992612

[B11] Chunchai T. Thunapong W. Yasom S. Wanchai K. Eaimworawuthikul S. Metzler G. et al. (2018). Decreased microglial activation through gut-brain axis by prebiotics, probiotics, or synbiotics effectively restored cognitive function in obese-insulin resistant rats. *J. Neuroinflamm.* 15:11. 10.1186/s12974-018-1055-2 29316965 PMC5761137

[B12] Coviello M. Barone D. Abate A. Geronimo A. Cassano G. D. Caiaffa V. et al. (2025). One-year follow-up cognitive decline after hip fracture surgery: the prognostic role of NSE and S100B biomarkers in elderly patients, a multicentric study. *J. Funct. Morphol Kinesiol*. 10:380. 10.3390/jfmk10040380 41133570 PMC12550907

[B13] De Felice F. G. Ferreira S. T. (2014). Inflammation, defective insulin signaling, and mitochondrial dysfunction as common molecular denominators connecting type 2 diabetes to Alzheimer disease. *Diabetes* 63 2262–2272. 10.2337/db13-1954 24931033

[B14] Erbil D. Riederer P. Lorke D. E. (2022). Repurposing GLP-1 receptor agonists and SGLT-2 inhibitors for Alzheimer’s disease. *Curr. Alzheimer Res.* 19 15–28.

[B15] Feinkohl I. Janke J. Slooter A. J. C. Winterer G. Spies C. Pischon T. (2023). Metabolic syndrome and the risk of postoperative delirium and postoperative cognitive dysfunction: a multi-centre cohort study. *Br. J. Anaesth*. 131 338–347. 10.1016/j.bja.2023.04.031 37344340

[B16] Feinkohl I. Janke J. Slooter A. J. C. Winterer G. Spies C. Pischon T. (2024). The association of plasma leptin, soluble leptin receptor and total and high-molecular weight adiponectin with the risk of perioperative neurocognitive disorders. *Am. J. Geriatr. Psychiatry* 32 1119–1129. 10.1016/j.jagp.2024.03.015 38637191

[B17] Feinkohl I. Winterer G. Pischon T. (2016). Obesity and post-operative cognitive dysfunction: a systematic review and meta-analysis. *Diabetes Metab. Res. Rev*. 32 643–651. 10.1002/dmrr.2786 26890984

[B18] Feinkohl I. Winterer G. Pischon T. (2017). Hypertension and risk of post-operative cognitive dysfunction (POCD): a systematic review and meta-analysis. *Clin. Pract. Epidemiol. Ment. Health* 13 27–42. 10.2174/1745017901713010027 28603544 PMC5447947

[B19] Feinkohl I. Winterer G. Pischon T. (2018). Associations of dyslipidaemia and lipid-lowering treatment with risk of postoperative cognitive dysfunction: a systematic review and meta-analysis. *J. Epidemiol. Commun. Health* 72 499–506. 10.1136/jech-2017-210338 29437865

[B20] Feng X. Degos V. Koch L. G. Britton S. L. Zhu Y. Vacas S. et al. (2013). Surgery results in exaggerated and persistent cognitive decline in a rat model of the Metabolic Syndrome. *Anesthesiology* 118 1098–1105. 10.1097/ALN.0b013e318286d0c9 23353794 PMC5530762

[B21] Florido-Santiago M. Pérez-Belmonte L. M. Osuna-Sánchez J. Barbancho M. A. Ricci M. Millán-Gómez M. et al. (2023). Assessment of long-term cognitive dysfunction in older patients who undergo heart surgery. *Neurologia* 38 399–404. 10.1016/j.nrleng.2020.12.005 37344096

[B22] Franceschi C. Campisi J. (2014). Chronic inflammation (inflammaging) and its potential contribution to age-associated diseases. *J. Gerontol. A Biol. Sci. Med. Sci*. 69 (Suppl. 1), S4–S9. 10.1093/gerona/glu057 24833586

[B23] Gheysen F. Poppe L. DeSmet A. Swinnen S. Cardon G. De Bourdeaudhuij I. et al. (2018). Physical activity to improve cognition in older adults: Can physical activity programs enriched with cognitive challenges enhance the effects? A systematic review and meta-analysis. *Int. J. Behav. Nutr. Phys. Act* 15:63. 10.1186/s12966-018-0697-x 29973193 PMC6032764

[B24] He X. Long G. Quan C. Zhang B. Chen J. Ouyang W. (2019). Insulin resistance predicts postoperative cognitive dysfunction in elderly gastrointestinal patients. *Front. Aging Neurosci*. 11:197. 10.3389/fnagi.2019.00197 31440156 PMC6694405

[B25] Heyer E. J. Mergeche J. L. Wang S. Gaudet J. G. Connolly E. S. (2015). Impact of cognitive dysfunction on survival in patients with and without statin use following carotid endarterectomy. *Neurosurgery* 77 880–887. 10.1227/NEU.0000000000000904 26308635

[B26] Huang Q. Du X. Ouyang W. Wang J. Liu X. (2025). Relationship between triglyceride-glucose index and all-cause mortality in older adults with sarcopenic obesity. *Metabol. Open* 27:100388. 10.1016/j.metop.2025.100388 40893912 PMC12398777

[B27] Hudetz J. A. Patterson K. M. Amole O. Riley A. V. Pagel P. S. (2011a). Postoperative cognitive dysfunction after noncardiac surgery: effects of metabolic syndrome. *J. Anesth*. 25 337–344. 10.1007/s00540-011-1137-0 21516370

[B28] Hudetz J. A. Patterson K. M. Iqbal Z. Gandhi S. D. Pagel P. S. (2011b). Metabolic syndrome exacerbates short-term postoperative cognitive dysfunction in patients undergoing cardiac surgery: results of a pilot study. *J. Cardiothorac. Vasc. Anesth*. 25 282–287. 10.1053/j.jvca.2010.06.008 20728380

[B29] Jan A. Shah M. Shah S. A. Habib S. H. Ehtesham E. Ahmed N. (2024). Melatonin rescues pregnant female mice and their juvenile offspring from high fat diet-induced Alzheimer disease neuropathy. *Heliyon* 10:e36921. 10.1016/j.heliyon.2024.e36921 39281480 PMC11395765

[B30] Jiang X. Ding L. Guo Y. Miao X. Zhao K. Chen L. et al. (2024). Association of metabolic syndrome and frailty with postoperative complications in older gastric cancer patients: a body composition perspective. *Cancer Med*. 13:e70194. 10.1002/cam4.70194 39315666 PMC11420831

[B31] Jiao X. H. Wan J. Wu W. F. Ma L. H. Chen C. Dong W. et al. (2024). GLT-1 downregulation in hippocampal astrocytes induced by type 2 diabetes contributes to postoperative cognitive dysfunction in adult mice. *CNS Neurosci. Ther*. 30:e70024. 10.1111/cns.70024 39218798 PMC11366448

[B32] Kostrzewska P. Kuca P. Witek P. Małyszko J. Madetko Alster N. Alster P. et al. (2025). SGLT-2 inhibitors in the prevention and progression of neurodegenerative diseases: a narrative review. *Neurol. Therapy* 14 2295–2312. 10.1007/s40120-025-00832-9 41071460 PMC12623600

[B33] Kotfis K. Szylińska A. Listewnik M. Brykczyński M. Ely E. W. Rotter I. (2019). Diabetes and elevated preoperative HbA1c level as risk factors for postoperative delirium after cardiac surgery: an observational cohort study. *Neuropsychiatr. Dis. Treat.* 15 511–521. 10.2147/NDT.S196973 30863073 PMC6388975

[B34] Li Y. L. Huang H. F. Le Y. (2021). Risk factors and predictive value of perioperative neurocognitive disorders in elderly patients with gastrointestinal tumors. *BMC Anesthesiol*. 21:193. 10.1186/s12871-021-01405-7 34281529 PMC8287702

[B35] Lin Y. Peng X. Lin X. Deng X. Liu F. Tao H. et al. (2022). Potential value of serum lipid in the identication of postoperative delirium undergoing knee/hip arthroplasty: the perioperative neurocognitive disorder and biomarker lifestyle study. *Front. Psychiatry* 13:870317. 10.3389/fpsyt.2022.870317 35492710 PMC9039337

[B36] Liu H. Chen J. Ling J. Wu Y. Yang P. Liu X. et al. (2025). The association between diabetes mellitus and postoperative cognitive dysfunction: a systematic review and meta-analysis. *Int. J. Surg*. 111 2633–2650. 10.1097/JS9.0000000000002156 39728730 PMC12372729

[B37] Liu L. Gong M. Liao G. Zhao W. Fu Q. (2025). [Hypertension exacerbates postoperative learning and memory impairment in rats possibly due to UCP2 downregulation-mediated mitochondrial dysfunction]. *Nan Fang Yi Ke Da Xue Xue Bao* 45 725–735. 10.12122/j.issn.1673-4254.2025.04.07 40294922 PMC12037294

[B38] Ma Y. Ji Y. Xu L. Li Z. Ge S. (2022). Obesity aggravated hippocampal-dependent cognitive impairment after sleeve gastrectomy in C57/BL6J mice via SIRT1/CREB/BDNF pathway. *Exp. Brain Res.* 240 2897–2906. 10.1007/s00221-022-06465-w 36114835

[B39] Machado-Fragua M. D. Fayosse A. Yerramalla M. S. van Sloten T. T. Tabak A. G. Kivimaki M. et al. (2022). Association of metabolic syndrome with incident dementia: role of number and age at measurement of components in a 28-year follow-up of the whitehall II cohort study. *Diabetes Care* 45 2127–2135. 10.2337/dc22-0206 35819815 PMC9472484

[B40] Mackey-Alfonso S. E. Butler M. J. Taylor A. M. Williams-Medina A. R. Muscat S. M. Fu H. et al. (2024). Short-term high fat diet impairs memory, exacerbates the neuroimmune response, and evokes synaptic degradation via a complement-dependent mechanism in a mouse model of Alzheimer’s Disease. *Brain Behav. Immunity* 121 56–69. 10.1016/j.bbi.2024.07.021 39043341 PMC12991061

[B41] Magariños A. M. McEwen B. S. (2000). Experimental diabetes in rats causes hippocampal dendritic and synaptic reorganization and increased glucocorticoid reactivity to stress. *Proc. Natl. Acad. Sci. U. S. A*. 97 11056–11061. 10.1073/pnas.97.20.11056 11005876 PMC27147

[B42] Mergenthaler P. Lindauer U. Dienel G. A. Meisel A. (2013). Sugar for the brain: the role of glucose in physiological and pathological brain function. *Trends Neurosci*. 36 587–597. 10.1016/j.tins.2013.07.001 23968694 PMC3900881

[B43] Mikami T. Kim J. Park J. Lee H. Yaicharoen P. Suidasari S. et al. (2021). Olive leaf extract prevents obesity, cognitive decline, and depression and improves exercise capacity in mice. *Sci. Rep*. 11:12495. 10.1038/s41598-021-90589-6 34127683 PMC8203715

[B44] Miller L. R. Bickel M. A. Vance M. L. Vaden H. Nagykaldi D. Nyul-Toth A. et al. (2024). Vascular smooth muscle cell-specific Igf1r deficiency exacerbates the development of hypertension-induced cerebral microhemorrhages and gait defects. *Geroscience* 46 3481–3501. 10.1007/s11357-024-01090-7 38388918 PMC11009188

[B45] Mullen J. T. Moorman D. W. Davenport D. L. (2009). The obesity paradox: body mass index and outcomes in patients undergoing nonbariatric general surgery. *Ann. Surg*. 250 166–172. 10.1097/SLA.0b013e3181ad8935 19561456

[B46] Muscat S. M. Butler M. J. Bettes M. N. DeMarsh J. W. Scaria E. A. Deems N. P. et al. (2024). Post-operative cognitive dysfunction is exacerbated by high-fat diet via TLR4 and prevented by dietary DHA supplementation. *Brain Behav. Immun*. 116 385–401. 10.1016/j.bbi.2023.12.028 38145855 PMC10872288

[B47] Neerland B. E. Krogseth M. Juliebø V. Hylen Ranhoff A. Engedal K. Frihagen F. et al. (2017). Perioperative hemodynamics and risk for delirium and new onset dementia in hip fracture patients; a prospective follow-up study. *PLoS One* 12:e0180641. 10.1371/journal.pone.0180641 28700610 PMC5503267

[B48] Newman M. F. Kirchner J. L. Phillips-Bute B. Gaver V. Grocott H. Jones R. H. et al. (2001). Longitudinal assessment of neurocognitive function after coronary-artery bypass surgery. *N. Engl. J. Med*. 344 395–402. 10.1056/NEJM200102083440601 11172175

[B49] Ni Y. Yang X. Yang Y. Zhang H. Peng S. (2025). Incidence and factors associated with postoperative delirium after primary total joint arthroplasty in older adults: a systematic review and meta-analysis. *Front. Med*. 12:1664605. 10.3389/fmed.2025.1664605 41200112 PMC12586022

[B50] Ntalouka M. P. Arnaoutoglou E. Vrakas S. Staikou C. Angelis F. A. Papadopoulos G. et al. (2022). The effect of type 2 diabetes mellitus on perioperative neurocognitive disorders in patients undergoing elective noncardiac surgery under general anesthesia. A prospective cohort study. *J. Anaesthesiol. Clin. Pharmacol*. 38 252–262. 10.4103/joacp.JOACP_292_20 36171952 PMC9511857

[B51] Palmer A. K. Xu M. Zhu Y. Pirtskhalava T. Weivoda M. M. Hachfeld C. M. et al. (2019). Targeting senescent cells alleviates obesity-induced metabolic dysfunction. *Aging Cell* 18:e12950. 10.1111/acel.12950 30907060 PMC6516193

[B52] Prasad S. Sajja R. K. Naik P. Cucullo L. (2014). Diabetes mellitus and blood-brain barrier dysfunction: an overview. *J. Pharmacovigil*. 2:125. 10.4172/2329-6887.1000125 25632404 PMC4306190

[B53] Puri S. Shaheen M. Grover B. (2023). Nutrition and cognitive health: a life course approach. *Front. Public Health* 11:1023907. 10.3389/fpubh.2023.1023907 37050953 PMC10083484

[B54] Qiu L. Q. Song J. L. Zhang L. C. Fan B. Li Q. Lu B. et al. (2025). Association between hyperlipidemia and postoperative delirium risk: a systematic review and meta-analysis. *Front. Aging Neurosci*. 17:1544838. 10.3389/fnagi.2025.1544838 40171385 PMC11959067

[B55] Rempe R. G. Hartz A. M. S. Bauer B. (2016). Matrix metalloproteinases in the brain and blood-brain barrier: versatile breakers and makers. *J. Cereb. Blood Flow Metab*. 36 1481–1507. 10.1177/0271678X16655551 27323783 PMC5012524

[B56] Saklayen M. G. (2018). The global epidemic of the metabolic syndrome. *Curr. Hypertens. Rep*. 20:12. 10.1007/s11906-018-0812-z 29480368 PMC5866840

[B57] Sándor ÁD. Sikos P. M. Varinot G. Kallinikos F. Mánfai C. Ifju M. et al. (2025). Association of diabetes with greater mid-term cognitive decline after carotid surgery. *Biomedicines* 13:2188. 10.3390/biomedicines13092188 41007750 PMC12467839

[B58] Santisteban M. M. Iadecola C. Carnevale D. (2023). Hypertension, neurovascular dysfunction, and cognitive impairment. *Hypertension* 80 22–34. 10.1161/HYPERTENSIONAHA.122.18085 36129176 PMC9742151

[B59] Saravia F. E. Revsin Y. Gonzalez Deniselle M. C. Gonzalez S. L. Roig P. Lima A. et al. (2002). Increased astrocyte reactivity in the hippocampus of murine models of type 1 diabetes: the nonobese diabetic (n.d.) and streptozotocin-treated mice. *Brain Res*. 957 345–353. 10.1016/s0006-8993(02)03675-2 12445977

[B60] Sebastian M. J. Khan S. K. Pappachan J. M. Jeeyavudeen M. S. (2023). Diabetes and cognitive function: an evidence-based current perspective. *World J. Diabetes* 14 92–109. 10.4239/wjd.v14.i2.92 36926658 PMC10011899

[B61] Shen H. Xiong Y. Liang Y. Liu Z. Guo L. Qin Y. et al. (2025). The effects of hyperlipidemia on postoperative cognitive dysfunction in patients undergoing laparoscopic gynecological tumor surgery. *Neuropsychiatr. Dis. Treat*. 21 259–269. 10.2147/NDT.S506570 39963123 PMC11831913

[B62] Shirvani F. Naji S. A. Davari E. Sedighi M. (2020). Early mobilization reduces delirium after coronary artery bypass graft surgery. *Asian Cardiovasc. Thorac. Ann*. 28 566–571. 10.1177/0218492320947230 32757652

[B63] Spitznagel M. B. Alosco M. Galioto R. Strain G. Devlin M. Sysko R. et al. (2014). The role of cognitive function in postoperative weight loss outcomes: 36-month follow-up. *Obes. Surg*. 24 1078–1084. 10.1007/s11695-014-1205-2 24570090 PMC4047156

[B64] Stanley M. E. Phillips R. K. III. Feng J. Shi G. Kant S. Sellke N. C. et al. (2025). The role of preoperative chronic hypertension in neurocognitive decline after cardiac surgery: a retrospective cohort study. *Braz. J. Cardiovasc. Surg.* 40:e20230470. 10.21470/1678-9741-2023-0470 39937691 PMC11813188

[B65] Stern J. H. Rutkowski J. M. Scherer P. E. (2016). Adiponectin, leptin, and fatty acids in the maintenance of metabolic homeostasis through adipose tissue crosstalk. *Cell Metab*. 23 770–784. 10.1016/j.cmet.2016.04.011 27166942 PMC4864949

[B66] Szeto H. H. (2014). First-in-class cardiolipin-protective compound as a therapeutic agent to restore mitochondrial bioenergetics. *Br. J. Pharmacol.* 171 2029–2050. 10.1111/bph.12461 24117165 PMC3976620

[B67] Tarantini S. Valcarcel-Ares N. M. Yabluchanskiy A. Fulop G. A. Hertelendy P. Gautam T. et al. (2018). Treatment with the mitochondrial-targeted antioxidant peptide SS-31 rescues neurovascular coupling responses and cerebrovascular endothelial function and improves cognition in aged mice. *Aging Cell* 17:e12731. 10.1111/acel.12731 29405550 PMC5847870

[B68] Toth P. Tarantini S. Csiszar A. Ungvari Z. (2017). Functional vascular contributions to cognitive impairment and dementia: mechanisms and consequences of cerebral autoregulatory dysfunction, endothelial impairment, and neurovascular uncoupling in aging. *Am. J. Physiol. Heart Circ. Physiol*. 312 H1–H20. 10.1152/ajpheart.00581.2016 27793855 PMC5283909

[B69] Travica N. Lotfaliany M. Marriott A. Safavynia S. A. Lane M. M. Gray L. et al. (2023). Peri-operative risk factors associated with post-operative cognitive dysfunction (POCD): an umbrella review of meta-analyses of observational studies. *J. Clin. Med*. 12:1610. 10.3390/jcm12041610 36836145 PMC9965885

[B70] Tucsek Z. Toth P. Tarantini S. Sosnowska D. Gautam T. Warrington J. P. et al. (2014). Aging exacerbates obesity-induced cerebromicrovascular rarefaction, neurovascular uncoupling, and cognitive decline in mice. *J. Gerontol. A Biol. Sci. Med. Sci*. 69 1339–1352. 10.1093/gerona/glu080 24895269 PMC4204615

[B71] Ungvari Z. Toth P. Tarantini S. Prodan C. I. Sorond F. Merkely B. et al. (2021). Hypertension-induced cognitive impairment: from pathophysiology to public health. *Nat. Rev. Nephrol*. 17 639–654. 10.1038/s41581-021-00430-6 34127835 PMC8202227

[B72] van Zuylen M. L. van Wilpe R. Ten Hoope W. Willems H. C. Geurtsen G. J. Hulst A. H. et al. (2022). Comparison of postoperative neurocognitive function in older adult patients with and without diabetes mellitus. *Gerontology* 69 189–200. 10.1159/000524886 35660665

[B73] Wang T. Sun G. Tao B. (2024). Updated insights into the NLRP3 inflammasome in postoperative cognitive dysfunction: emerging mechanisms and treatments. *Front. Aging Neurosci*. 16:1480502. 10.3389/fnagi.2024.1480502 39411285 PMC11474915

[B74] Ward R. Li W. Abdul Y. Jackson L. Dong G. Jamil S. et al. (2019). NLRP3 inflammasome inhibition with MCC950 improves diabetes-mediated cognitive impairment and vasoneuronal remodeling after ischemia. *Pharmacol. Res*. 142 237–250. 10.1016/j.phrs.2019.01.035 30818045 PMC6486792

[B75] Windmann V. Spies C. Knaak C. Wollersheim T. Piper S. K. Vorderwülbecke G. et al. (2019). Intraoperative hyperglycemia increases the incidence of postoperative delirium. *Minerva Anestesiol.* 85 1201–1210. 10.23736/S0375-9393.19.13748-0 31486622

[B76] Xiong B. Shi Q. Fang H. (2016). Dexmedetomidine alleviates postoperative cognitive dysfunction by inhibiting neuron excitation in aged rats. *Am. J. Transl. Rese.* 8 70–80. PMC4759417 27069541

[B77] Yang C. Zhu B. Ding J. Wang Z. G. (2014). Isoflurane anesthesia aggravates cognitive impairment in streptozotocin-induced diabetic rats. *Int. J. Clin. Exp. Med.* 7 903–910. 24955160 PMC4057839

[B78] Yang P. Fan Y. Tang W. (2025). Correlation of intraoperative blood pressure variability and postoperative delirium in elderly hip fracture surgery. *Sci. Rep.* 15:15007. 10.1038/s41598-025-00019-0 40301442 PMC12041256

[B79] Yao F. Liu L. Chen X. Zhang J. Hao Z. He Z. (2025). Triglyceride-glucose index and postoperative delirium: a retrospective study exploring a lipid-related marker in neuropsychiatric risk stratification after gastric surgery. *Lipids Health Dis*. 24:361. 10.1186/s12944-025-02794-1 41233811 PMC12613469

[B80] Zeng J. Li N. Ma C. Song Y. Tian W. Gao Y. et al. (2025). Association between triglyceride-glucose index and postoperative delirium following cardiac surgery: a multicenter retrospective analysis. *Lipids Health Dis*. 24:330. 10.1186/s12944-025-02769-2 41102683 PMC12533457

[B81] Zhang M. Zhu Y. H. Zhu Z. Q. (2022). Research advances in the influence of lipid metabolism on cognitive impairment. *Ibrain* 10 83–92. 10.1002/ibra.12018 38682015 PMC11045198

[B82] Zhang X. Yan X. Gorman J. Hoffman S. N. Zhang L. Boscarino J. A. (2014). Perioperative hyperglycemia is associated with postoperative neurocognitive disorders after cardiac surgery. *Neuropsychiatr. Dis. Treat.* 10 361–370. 10.2147/NDT.S57761 24570589 PMC3933727

[B83] Zhang X. P. Liu Y. R. Chai M. Yang H. T. Wang G. Han M. et al. (2019). High-fat treatment prevents postoperative cognitive dysfunction in a hyperlipidemia model by protecting the blood-brain barrier via Mfsd2a-related signaling. *Mol. Med. Rep*. 20 4226–4234. 10.3892/mmr.2019.10675 31545471 PMC6797931

[B84] Zhao D. Li J. Yang R. Xu G. (2020). Effects of stage I hypertension on the recovery of early postoperative attention network function in elderly patients undergoing elective hip or knee arthroplasty surgery. *Turk. J. Med. Sci*. 50 37–43. 10.3906/sag-1902-58 31655525 PMC7080347

[B85] Zhao J. Liang G. Hong K. Pan J. Luo M. Liu J. et al. (2022). Risk factors for postoperative delirium following total hip or knee arthroplasty: a meta-analysis. *Front. Psychol*. 13:993136. 10.3389/fpsyg.2022.993136 36248575 PMC9565976

[B86] Zhao Y. Zhong K. Zheng Y. Xia X. Lin X. Kowark A. et al. (2024). Postoperative delirium risk in patients with hyperlipidemia: a prospective cohort study. *J. Clin. Anesth*. 98:111573. 10.1016/j.jclinane.2024.111573 39094442

[B87] Zhao Z. Yao M. Wei L. Ge S. (2020). Obesity caused by a high-fat diet regulates the Sirt1/PGC-1α/FNDC5/BDNF pathway to exacerbate isoflurane-induced postoperative cognitive dysfunction in older mice. *Nutr. Neurosci*. 23 971–982. 10.1080/1028415X.2019.1581460 30794116

[B88] Zhu S. H. Ji M. H. Gao D. P. Li W. Y. Yang J. J. (2014). Association between perioperative blood transfusion and early postoperative cognitive dysfunction in aged patients following total hip replacement surgery. *Ups J. Med. Sci*. 119 262–267. 10.3109/03009734.2013.873502 24345210 PMC4116766

[B89] Zhu Y. He Y. Yang H. Gao Y. Wang Y. Liu P. et al. (2025). Semaglutide ameliorates diabetes-associated cognitive dysfunction in mouse model of type 2 diabetes. *PLoS One* 20:e326897. 10.1371/journal.pone.0326897 40608828 PMC12225814

